# Complement activation during cardiopulmonary bypass and association with clinical outcomes

**DOI:** 10.1002/jha2.371

**Published:** 2022-01-13

**Authors:** Rengina Kefalogianni, Farah Kamani, Mihaela Gaspar, TC Aw, Jackie Donovan, Mike Laffan, Matthew C. Pickering, Deepa J. Arachchillage

**Affiliations:** ^1^ Department of Immunology and Inflammation Imperial College London London UK; ^2^ Department of Haematology Royal Brompton Hospital London UK; ^3^ Department of Anesthesia and Critical Care Royal Brompton Hospital London UK; ^4^ Department of Biochemistry Royal Brompton Hospital London UK; ^5^ Centre for Haematology Department of Immunology and Inflammation Imperial College London London UK

**Keywords:** anaphylatoxins, bleeding, cardiopulmonary bypass, complement, heparin, protamine sulphate

## Abstract

In this prospective, single‐centre observational study of 30 patients undergoing cardiopulmonary bypass (CPB), the effect of unfractionated heparin (UFH), CPB surgery and protamine sulphate on complement and on post‐operative blood loss were assessed. Although C3 and C4 levels decreased significantly immediately following the administration of UFH, C3a, C5a, Bb fragment and SC5b‐9 remained unchanged. During CPB, C3 and C4 continued to fall whilst both alternative and classical pathways activation markers, Bb, C3a, C5a and SC5b‐9 increased significantly. Protamine sulphate had no effect on classical pathway components or activation markers but decreased alternative pathway activation marker Bb. Over the 12–24 h post‐surgery, both classical and alternative pathway activation markers returned to baseline, whilst C3 and C4 levels increased significantly but not to baseline values. Total drain volume 24 h after the surgery showed a moderate inverse correlation with post‐protamine C3 (*r* = −0.46, *p* = 0.01) and C4 (*r* = −0.57, *p* = 0.0009) levels, whilst a moderate positive correlation was observed with post‐protamine C3a (*r* = 0.46, *p* = 0.009), C5a (*r* = 0.37, *p* = 0.04) and SC5b‐9 (*r* = 0.56, *p* = 0.001) levels but not with Bb fragment (*r* = 0.25, *p* = 0.17). Thus, inhibition of complement activation may be a therapeutic intervention to reduce post‐operative blood in patients undergoing CPB.

## 1 INTRODUCTION

Cardiopulmonary bypass (CPB) is an extracorporeal circuit that provides circulatory and respiratory support during cardiac surgery [1]. During CPB a systemic inflammatory response syndrome (SIRS) is induced [2], and production of several pro‐inflammatory mediators is increased [3] with the activation of complement activation occurring throughout CPB [4]. Rapid and excessive activation of the complement system may cause tissue damage and increased vascular permeability. Furthermore, it is often linked with the pathogenesis of multiple organ failure via granulocyte aggregation [5]. Complement activation occurs via the classical, alternative or lectin pathway all leading to the generation of C3‐convertase, an enzymatic complex [6].

Elevated complement activation markers including C3a, C5a and SC5b‐9 have been frequently observed during and after CPB [7, 8]. Complement activation during CPB occurs mainly via the alternative complement pathway due to the direct adhesion of C3 to the extracorporeal bypass circuit [7, 9]. In addition, plasmin, produced as a result of contact activation initiated by tissue injury, has been implicated in complement activation via direct activation of C3 [10, 11]. Some studies reported complement activation through the lectin pathway during CPB [12]. In a study with 185 children with congenital heart disease undergoing surgical correction with the use of CPB, changes in serum levels of mannose binding lectin and activities of MBL‐MBL‐associated serine protease (MASP)‐1 and MBL‐MASP‐2 complexes were assessed immediately before, and during the surgery, throughout the first postoperative day and at discharge from the hospital. Decreases in MBL and MBL‐MASP complexes were observed in all samples, correlating with a decrease in C4 and increase in C4a, confirming activation of the lectin pathway [12].

Complement activation has been linked with post‐operative complications including organ failure and haemodynamic instability [13, 14, 15, 16]. Evidence suggests pro‐inflammatory cytokine release can interfere with myocardial contractility [17, 18]. Furthermore, a study including 116 patients undergoing CPB showed that increased C3a levels were associated with renal and pulmonary dysfunction, abnormal bleeding and overall morbidity [15].

Several strategies have been used to reduce the impact of complement activation in patients undergoing CPB and cardiac surgery [19]. CPB circuits coated with a layer of heparin molecules have been proven to improve the biocompatibility of the bypass circuit: studies reported that C3a and SC5b‐9 levels were significantly lower in patients undergoing CPB with heparin‐coated circuits compared to control group, consistent with reduced activation [8]. In addition to heparin‐coated circuits reducing complement activation, they have been shown to improve outcomes in terms of postoperative recovery of the patient [9]. During cardiac surgery, blood contact with the extracorporeal surface of the bypass circuit increases the risk of blood clot formation [20]. To minimise this risk, unfractionated heparin (UFH) is administered to patients during cardiac surgery [21]. At CPB termination, the effects of UFH are reversed to restore haemostasis and reduce post‐operative bleeding. This is achieved by the administration of protamine sulphate, a polycationic alkaline molecule that binds to UFH producing a biologically inactive dense precipitate. Previous findings indicate that classical complement pathway activation is specifically induced by the heparin–protamine complex formation during CPB [21, 22, 23, 24]. As heparin has anti‐inflammatory and anti‐complement effects [25], the use of high dose UFH during CPB may reduce the complement activation in a dose‐dependent manner.

In this study we assessed the effect of UFH, CPB surgery and protamine sulphate on complement, complement activation markers and the effect of complement activation on post‐operative blood loss and clinical outcomes.

## 2 PATIENTS AND METHODS

### 2.1 Study design and participants

This was a prospective single‐centre pilot observational study conducted at a tertiary referral centre for cardiothoracic surgery in the United Kingdom (UK). The study complies with the principles laid down in the Declaration of Helsinki and was approved by the research ethics committee (REC), Health Research Authority and Health and Care Research Wales (REC reference 18/LO/2158). Thirty adult patients, undergoing their first CPB for repair of atrial septal defects (ASD) (*n* = 4) or tissue mitral valve replacement (*n* = 26) were included. All patients had elective surgery. Patients with baseline platelet count of <100 10^9^/l, or platelet dysfunction, receiving heparin or antiplatelet treatment prior to surgery or patients with concomitant aortic stenosis were excluded. All participants provided written informed consent prior to surgery.

### 2.2 CPB

All study participants underwent the same CPB technique during the cardiac surgery. We use CPB circuits manufactured by Maquet Ltd (Getinge) (Sunderland, Tyne & Wear, UK). The components of the circuit are coated with Softline (polymer comprised of hydrophilic and hydrophobic areas, reducing surface tension on the contact surfaces), whilst tubing is uncoated. The standard adult circuit priming volume of approximately 1200 ml (∼250 ml of Isoplex and 950 ml Hartmann's solution) was used for all patients, and haemoconcentration or ultra‐filtration was not performed. In our routine clinical practice, UFH 300–400 U/kg is administered aiming for an activated clotting time (ACT) of greater than 480 s prior to the CPB. However, it is known that the ACT poorly correlates with the plasma heparin level and does not accurately predict the protamine sulphate dose needed for reversal. Therefore, for this study, heparin anti‐Xa level was used to assess UFH presence and effect. UFH 300–400 IU/kg was administered intravenously to each patient initially, and further doses were given if the heparin anti‐Xa level was <4.0 u/ml. A standard dose of UFH 10,000 units was administered to all patients at the priming of the CPB followed by a median dose of 30,000 units (range 15,000–45,000 units) of UFH during the CPB. At CPB termination, protamine sulphate was administrated intravenously at a 1:1 ratio in keeping with 1 mg of protamine sulphate for 100 units of UFH administered (a median dose of 400 mg [range 300–550 mg]). All patients survived the procedure and had a normal recovery. It is our standard practice to administer tranexamic acid to all patients undergoing CPB surgery to reduce bleeding unless there is a contraindication to i.

### 2.3 Blood sample timing, processing and laboratory analyses

Venous blood samples were obtained from each participant at the following time points: Prior to administration of intravenous (IV) UFH (cannulation at the anaesthetic room as the baseline), 30 min post‐administration of IV UFH, prior to the administration of IV protamine sulphate following completion of CPB, 15 min (+/−5) post‐administration of IV protamine sulphate, 12 h and 24 h post‐CPB (Table 1). All patients had CPB initiated at the time post‐heparin samples were taken to assess the complement markers and other laboratory makers. Complement components and activation markers were assessed on all samples. Additionally, effect of post‐surgical inflammatory response on complement and their activation markers were assessed on samples taken at 12 h and 24 h post‐surgery.

**TABLE 1:** Sample collection timing in relation to CPB

	Time point	Sample name
**Day 0**	Pre‐IV Heparin (baseline) Cannulation at the anaesthetic room	Pre‐heparin
**Day 0**	Post‐IV heparin (30–60 min following 300–400 IU/kg of UFH administered intravenously)	Post‐heparin
**Day 0**	Pre‐protamine (15–30 min prior to administering protamine sulphate)	Pre‐protamine sulphate
**Day 0**	Post‐protamine (15 min (+/−5) following administration of protamine sulphate with1: 1 ratio in keeping with 1 mg of protamine sulphate for 100 units of UFH.)	Post‐protamine sulphate
**Day +1**	12 h post‐op (12 h following the end of CPB)	12 h post‐OP
**Day +1**	24 h post op (24 h following the end of CPB)	24 h post‐OP

Abbreviations: CPB, cardiopulmonary bypass; OP, operation; UFH, unfractionated heparin.

Blood samples were collected into tubes (Vacutainer Plus, Becton Dickinson, Franklyn Lakes USA), containing 0.109 M trisodium citrate (9:1 ratio) for heparin anti‐Xa level. Samples for complement activation markers were collected into potassium ethylenediaminetetraacetic acid tubes, centrifuged at 2000 g for 10 min to separate plasma and kept at −80°C until further analysis.

### 2.4 Anti‐Xa assay

A chromogenic liquid anti‐Xa assay (Werfen, Warrington, Cheshire, UK) was performed for quantitative determination of UFH activity in patient's plasma using an ACL TOP 500 machine (Werfen, Warrington, Cheshire, UK).

### 2.5 C3 and C4 level measurement

Plasma C3 and C4 were measured using an immunoturbidimetric technique on the AU680 analyser (Beckman Coulter, High Wycombe, UK). The reagent containing latex‐enhanced antibodies to C3 or C4 forms immune complexes with C3 or C4 in the plasma, which scatter light in proportion to their size, shape and concentration. The measurement of the decrease in light transmitted through particles suspended in solution, as a result of complex formation, is proportional to the amount of analyte present in the sample.

### 2.6 Complement activation assays

C3a desArg, C5a, Bb and sC5b‐9 were detected by sandwich enzyme‐linked immunosorbent assay (ELISA): complement C3a desArg human ELISA kit (cat. no. ab133037; Abcam, Cambridge, MA, USA), human complement C5a ELISA kit (cat. no. ab193695; Abcam, Cambridge, MA, USA), MicroVue Bb plus fragment EIA (cat. no. A027; Quidel Crop, Pathway diagnostics Ltd, Dorking, UK) and MicroVue SC5b‐9 Plus EIA (cat. no. A029; Quidel Crop, Pathway diagnostics Ltd, Dorking, UK). Absorbance was measured at 450 nm (NS‐100 Nano Scan Microplate Reader), and concentrations were determined using the commercial standard curves provided with the kits.

### 2.7 Statistical analysis

Data are presented as percentages for categorical data, median and ranges for continuous data. Difference between each study time point was assessed using multiple comparison of two‐way Analysis of Variance (ANOVA) (Bonferroni and Sidak multiple comparisons). Correlation between the complement markers and heparin anti‐Xa levels and the duration of surgery was assessed using Spearman correlation. Analyses were performed using GraphPad Prism version 9.3.1 (GraphPad Software, Inc. La Jolla, USA). Two‐tailed *p* < 0.05 were considered statistically significant.

## 3 RESULTS

Median age of the study participants was 62 years, and 56.7% (17/30) were male. Clinical and laboratory characteristics of 30 patients included in the study are presented in Table 2. Cell‐saver was used for all patients, and the median volume of cell salvage was 2211 ml (range 1126–6255 ml). Median volume of cells reinfused was 650 ml (264–1500 ml). None of the patients had autologous blood harvest.

**TABLE 2:** Demographics and laboratory characteristics of 30 patients had cardiopulmonary bypass at baseline (pre‐heparin)

Variable	% or median	Interquartile range	Normal range
Male	56.7% (17/30)		
Female	43.3% (13/30)		
Age (years)	62	22.5	
Height (m)	1.77	0.21	
Weight (kg)	84	37.2	
Body mass index (BMI)	26.3	6.9	
EuroSCORE2	1.32	1.8	
CPB duration (hours)	2.05	0.9	
Duration of surgery (hours)	4.1	1.5	
Haemoglobin (g/L)	136	18.5	121–172
Baseline platelet count (x10^9^/L)	192	92	150–450
APTT (seconds)	28.2	3.45	26–36
PT (seconds)	13.6	2.1	10.2–13.2
Fibrinogen (g/L)	2.6	1.25	1.5–4.5
C‐reactive protein (mg/L)	3,0	5.5	0–10
Albumin (g/L)	43	5	35–55
Total protein (g/L)	72.5	7.25	60–83
Baseline Creatinine Clearance (ml/min) (using Cockcroft–Gault equation)	100.9	54.89	88–137

Abbreviations: APTT, activated partial thromboplastin time; CPB, cardiopulmonary bypass; PT, prothrombin time.

### 3.1 Changes in plasma C3 and C4 levels during CPB

**FIGURE 1:**
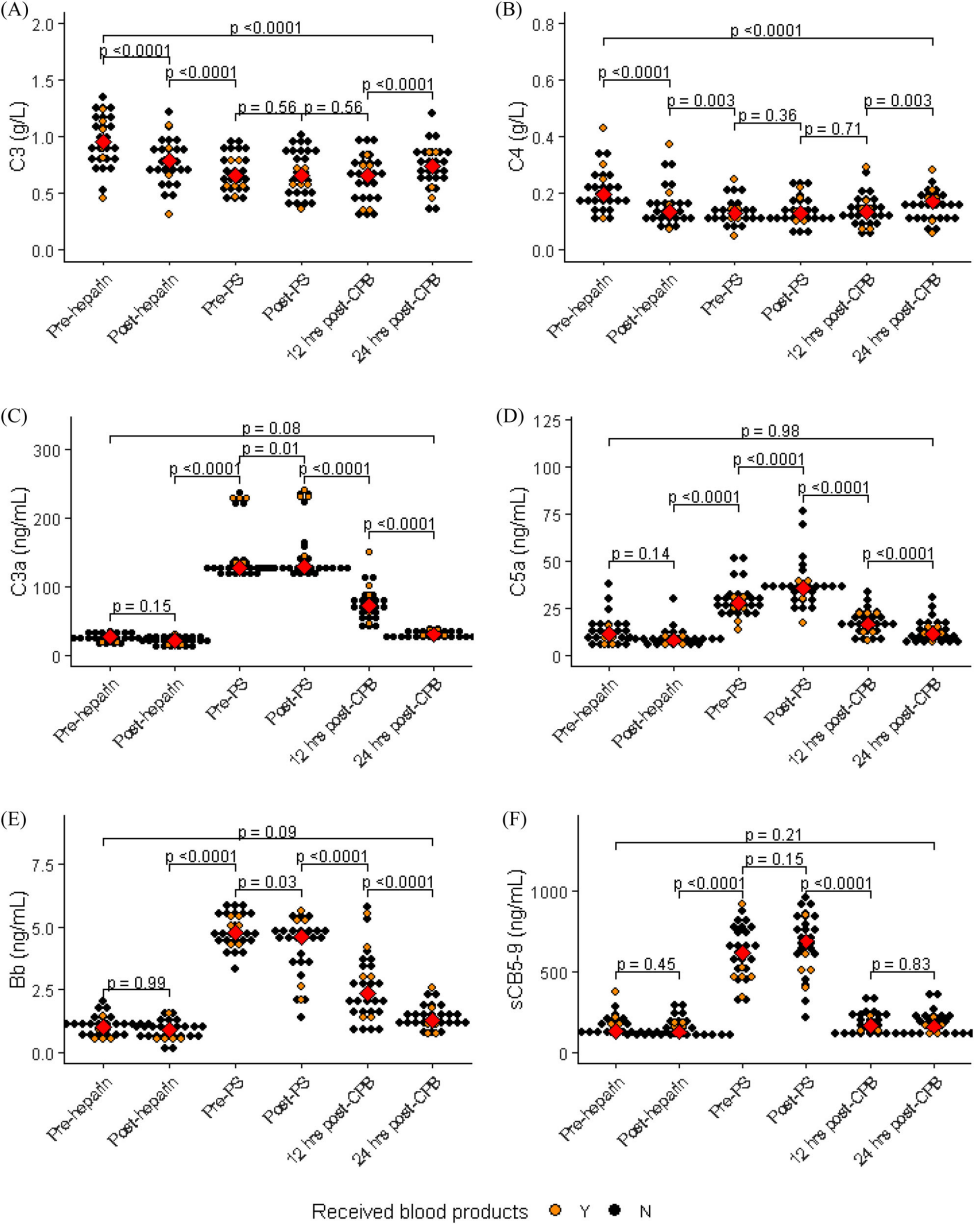
Plasma concentrations of C3 (A) and C4 (B) complement factors, complement activation markers C3a (C), C5a (D), Bb fragment (E) and sC5b‐9 (F) (in 30 patients undergoing cardiopulmonary bypass. Blood samples were analysed at six study points (baseline [pre‐heparin], pre‐protamine sulphate [PS], post‐protamine sulphate [PS], 12 and 24 h post‐CPB): Heparin: unfractionated heparin, protamine: protamine sulphate. Red squares indicate median, orange circles indicate complement components and activation markers of the patients who received blood components

**FIGURE 2:**
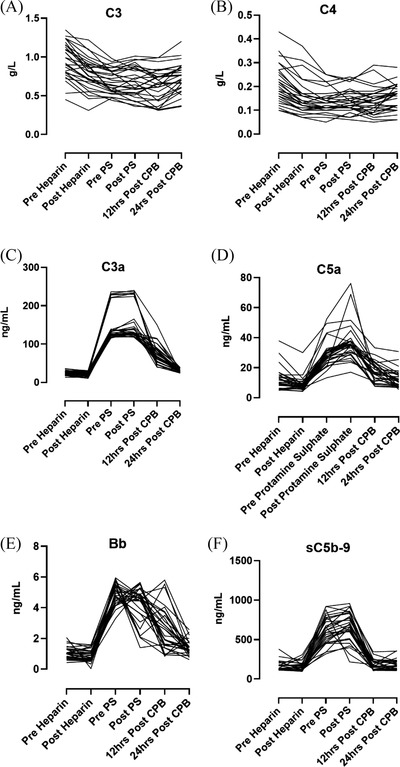
Changes of C3, C4, C3a, C5a, Bb fragment and sC5b‐9 levels over time from baseline (pre‐heparin) to 24 h post‐cardiopulmonary bypass (CPB)

Administration of UFH caused a significant fall in C3 plasma levels (*p* < 0.0001): pre heparin (median 1 g/L [range 0.45–1.35]) versus post‐heparin (0.8 g/L [0.31–1.22]). C3 levels continued to decrease during CPB and reached a nadir at the end of surgery. Protamine sulphate had no effect on C3 (0.6 g/L [0.36–1.0] vs. 0.6 g/L [0.31‐0.99], *p* = 0.5). C3 levels gradually increased over the next 12–24 h but remained significantly lower than baseline suggesting it takes more than 24 h post‐surgery for C3 levels to return normal levels (Figure 1A). Changes in C4 followed a similar pattern to C3. A significant decrease (*p* < 0.0001) in C4 levels was noted following administration of UFH. C4 levels continued to decrease (*p* = 0.003) during the surgery, and protamine sulphate had no effect on C4 levels. As with C3, C4 levels gradually increased over the next 12–24 h but remained significantly lower than baseline level (Figure 1B). Changes of C3 and C4 levels over time from baseline (pre‐heparin) to 24 h post‐CPB are shown in Figure 2A,B, respectively.

### 3.2 Changes in C3a and C5a levels during CPB

In contrast to C3 and C4, administration of UFH did not change C3a levels significantly (*p* = 0.15): pre‐heparin (median 25.5 ng/ml [range 13.1–36.1]) versus post‐heparin (21.2 ng/ml [10.2–30.7]). However, during the surgery, C3a levels were significantly increased (*p* < 0.0001), and there was a statistically significant rise after protamine sulphate. Over the next 12 h, there was a significant decrease in C3a levels with levels returning to baseline levels (before heparin administration) by 24 h post‐surgery (Figure 1C).

Similarly, UFH had no significant effect on C5a plasma levels (*p* = 0.14): pre heparin (12.4 ng/ml [0.99–37.9]) versus post‐heparin (8.8 ng/ml [4.1–29.9]). During the surgery, C5a levels increased significantly and continued to rise post‐protamine sulphate suggesting that protamine sulphate does not influence C5 activation. Following surgery, over the next 12–24 h, C5a levels gradually fall reaching baseline values by 24 h post‐surgery (Figure 1D). Changes of C3a and C5a levels over time from baseline (pre‐heparin) to 24 h post‐CPB are shown in Figure 2C,D respectively.

### 3.3 Changes in Bb during CPB

The alternative pathway activation marker Bb also followed a similar pattern to the anaphylatoxins (C3a, C5a), showing no significant difference between pre‐ and post‐UFH (*p* = 0.99): pre‐heparin levels 1.1 um/ml (0.42–2.0) versus post‐heparin levels 0.89 ug/ml (0.03–1.58). During the surgery, Bb levels significantly increased (*p* < 000.1) compared to post‐heparin and reached peak values prior to the administration of protamine sulphate. The administration of protamine sulphate was followed by a significant fall in Bb fragment levels, and the levels continued to fall over the next 12–24 h reaching baseline values by 24 h post‐surgery (Figure 1E). Changes of Bb fragments levels over time from baseline (pre‐heparin) to 24 h post‐CPB are shown in Figure 2E.

### 3.4 Changes in sC5b‐9 during CPB

The sC5b‐9 levels followed a similar pattern to the anaphylatoxins (C3a, C5a) remaining at similar levels (*p* = 0.42) before and after UFH administration ([range 112.1–230.6 ng/ml, mean 151.1 ng/ml], [range 96.2–307.9 ng/ml, mean 155.4 ng/ml], respectively). During surgery, sC5b‐9 increased significantly (*p* < 0.0001). However, protamine sulphate administration did not result in any significant change in sC5b‐9 (*p* = 0.15). Twelve hours after surgery, sC5b‐9 levels had decreased significantly (median 179.1 ng/ml [103.7–344.5 ng/ml] *p* = 0.0001) compared to post‐protamine sulphate, returning to baseline level by 24 h post‐surgery (Figure 1F). Changes of sC5b‐9 level over time from baseline (pre‐heparin) to 24 h post ‐CPB are shown in Figure 2F.

### 3.5 Correlation between complement components, activation markers and heparin anti‐Xa levels and the duration of the CPB prior to administration of protamine sulphate

**FIGURE 3:**
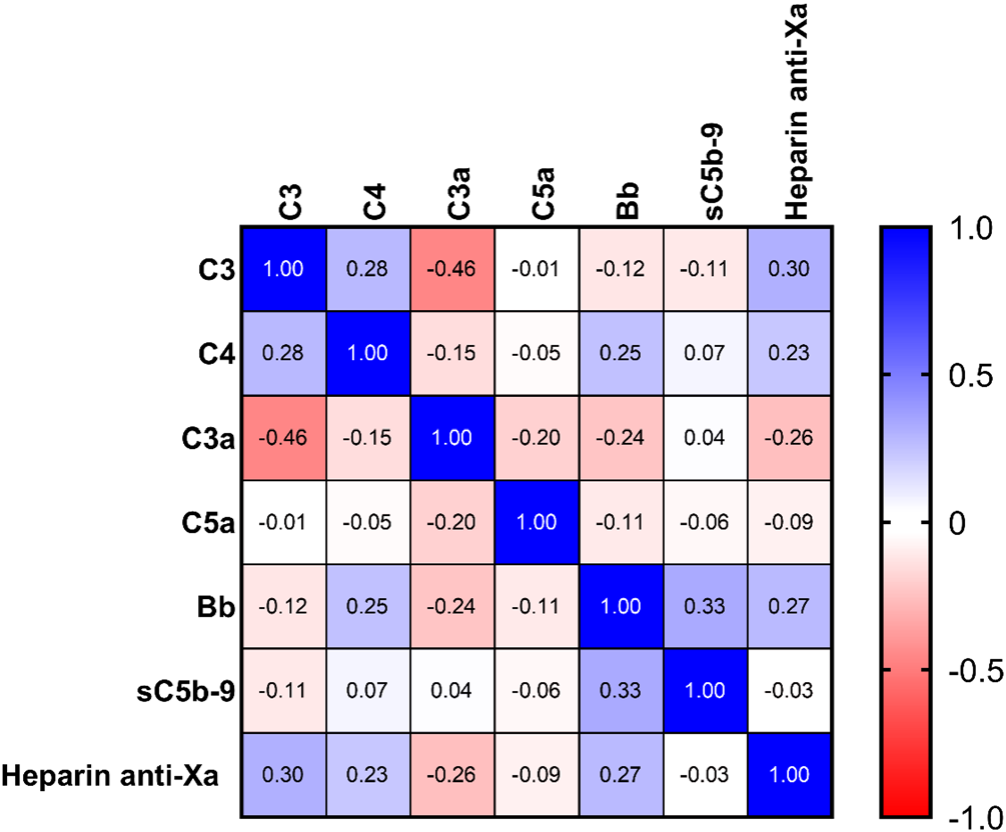
Correlations between C3, C4, C3a C5a, Bb fragment and the heparin anti‐Xa levels in samples taken prior to administration of protamine sulphate (pre‐protamine sulphate)

As heparin has anti‐complement effect, and longer duration of CPB would be expected to cause higher levels of complement activation, correlation between complement components or activation markers with heparin level and the duration of CPB was assessed. The correlations between complement components and activation markers with heparin levels (heparin anti‐Xa) in samples taken prior to the administration of protamine sulphate were not significant (Figure 3). Similarly, there was no correlation between CPB duration and the complement markers. Median duration of the CPB was 2.05 h (range 1.24–3.73 h). As expected, there was a significant negative correlation between C3 and C3a levels (*r* = −0.46, *p* = 0.01); however, there was no correlation between C3, C4 or complement activation markers (Figure 3). The median intra‐operative ACT had no correlation with anti‐Xa level at the Pre‐ protamine sulphate time point; Spearman correlation *r* = ‐ 0.03590, *p* = 0.86. Furthermore, as with heparin anti‐Xa levels, no correlation was seen between pre‐protamine sulphate ACT and the complement components or activation makers.

### 3.6 Effects of complement components and activation markers on clinical outcomes and use of blood products

**FIGURE 4:**
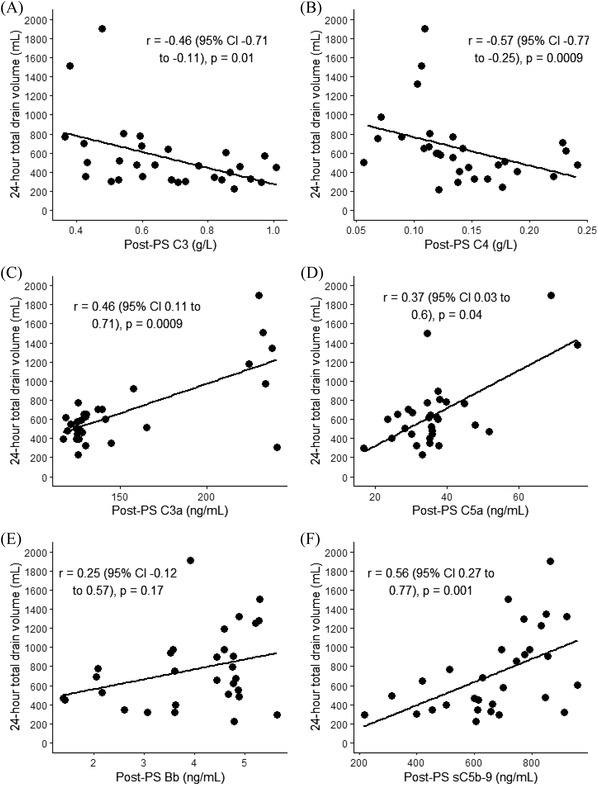
Correlation between post‐protamine C3, C4, C3a, C5a, Bb fragment and sC5b‐9 levels and blood loss following cardiopulmonary bypass as measured by total drain volume within 24 h of post‐surgery

Complement activation and the resulting inflammatory response may be an important mechanism for multisystem organ injury in patients undergoing CPB [19]. As complement and coagulation systems are closely linked, the correlation between complement components or activation markers with 24 h blood loss was assessed. Furthermore, clinical outcomes such as organ failure requiring additional support were also assessed. None of the patients developed renal failure or pulmonary dysfunction requiring ventilatory support. Bleeding was assessed at 24 h post‐operative by measuring the drain volume and compared to complement protein levels measured at the post‐protamine sulphate time point. Median 24‐h total drain volume was 587 ml (range 225–1900 ml). Blood loss from surgical drains in the first 24 h postoperatively was used to determine post‐operative bleeding as none of the patients required return to theatre for surgical bleeding. Total drain volume 24 h after the surgery showed a moderate inverse correlation with post‐protamine C3 (*r* = −0.46, *p* = 0.01, and C4 (*r* = −0.57, *p* = 0.0009) levels, whilst a moderate positive correlation was observed with post‐protamine C3a (*r* = 0.46, *p* = 0.009), C5a (*r* = 0.37 p = 0.04) and SC5b‐9 (*r* = 0.56, *p* = 0.001) levels, but no correlation was seen with Bb fragment (*r* = 0.25, *p* = 0.17) (Figure 4). Of 30 patients included in this study, only seven patients received any form of allogenic blood products. C3, C4 levels and complement activation markers (C3a, C5a, Bb fragment and sCB5‐9) of the seven patients who received blood products are highlighted in Figure 1. There were no outliers, and as the number of patients who received blood products was small, no further analysis was performed.

### 3.7 Assessment of plasma factor dilution

**FIGURE 5:**
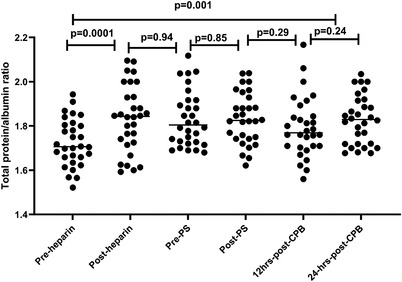
Total protein/albumin ratio to assess the plasma dilution during study period

Throughout bypass surgery, blood is considerably diluted which may influence complement levels. Plasma dilution was assessed using total protein/albumin ratio measured in samples taken at the same time points for complement assessment (Figure 5). As expected, there was a significant dilution in plasma pre‐ and post‐heparin (*p* = 0.0001), and this dilution remained significant compared to baseline (pre‐heparin) through the CPB and did not return to normal even at 24 h post‐CPB. However, there were no differences between post‐heparin and pre‐protamine sulphate, pre‐protamine sulphate and post‐protamine sulphate, post‐ protamine sulphate and 12 h post‐CPB or 12 h post‐CPB and 24 h post‐CPB (Figure 5). Plasma dilution factor was used to correct the measured values of complement components and the activation markers.

## 4 DISCUSSION

In this prospective single‐centre pilot observational study with patients undergoing CPB for correction of ASD or tissue mitral valve replacement, there was no difference in the classical or alternative pathways activation markers immediately following the administration of UFH but a significant rise in these markers (C3a, C5a sC5b‐9 and Bb fragment) during CPB, with gradual reduction in the level over 12–24 h post‐CPB. However, sC5b‐9 remained unchanged following protamine sulphate, although C3a and C5a levels continued to rise following the administration of protamine sulphate, whilst Bb fragment levels fell.

There are substantial data on the changes in complement activation during cardiac surgery, including CPB and extracorporeal membrane oxygenation. However, these studies have largely focused on the effect of complement activation in cardiac events such as myocardial infarction or myocardial contractility and mortality following the cardiac surgery [26, 27, 28]. Only a few studies have specifically assessed the relationship with post‐operative bleeding [29, 30] and, even then, those studies have considered the role of specific complement components rather than the overall assessment of the complement system provided in this report. This study provides prospective and systematic assessment of the complement components and activation markers in both classical and alternative pathways to provide a complete picture of the processes involved in determining the important clinical outcomes especially post‐operative bleeding. This fresh insight draws attention to an area in a CPB that warrants further assessment to determine if complement inhibition in patients undergoing CPB can reduce the post‐operative blood loss.

The significant reduction in C4 levels (Figure 1B) 30 min after the administration of heparin might suggest C4 cleavage by C1s enzyme; although we did not measure split of C4. However,there was no rise in C3a. Although these results may suggest that the fall in C3 and C4 levels is due to formation of complexes with heparin rather than activation, it is possible that the fall in C3 and C4 levels could be due to haemodilution and/or contact with the oxygenator and the circuit rather than due to any effect from heparin. However, results were corrected for dilution using total protein/albumin ratio as a plasma dilution factor. Additionally, immunological changes associated with CPB were already in process which could contribute to the observed lack of difference in the levels of complement activation markers following UFH (counterbalancing effects of the immunological activation of complement and anti‐complement effect of UFH). Therefore, it was practically impossible to isolate the effects on C3 and C4 levels from individual components.

Due to the anti‐complement effect of heparin, a negative correlation coefficient was expected when assessing the relationship between complement activation marker levels and heparin anti‐Xa levels. However, no significant correlation was observed between any of the complement markers and heparin anti‐Xa levels (Figure 3). It is therefore difficult to determine whether UFH has an effect independent of the effect of CPB, and these results do not reject the possibility of a relationship between complement activation marker levels and heparin anti‐Xa level which may be overshadowed by the surgery and CPB effect. As expected, there was a significant negative correlation between C3 level and C3a levels in samples taken prior to the administration of protamine sulphate. We did not observe correlation between C3 and C4 levels with other complement activation markers or especially a correlation between C3a and C5a or sC5b‐9 or Bb fragment. The observed lack of correlation between these markers could be due to variation in the complex interaction between CPB and heparin in individual patients. Interestingly, total drain volume 24 h after the surgery showed an inverse correlation with post‐protamine C3 and C4 levels, whilst a moderate positive correlation was observed with post‐protamine C3a, C5a and SC5b‐9 levels (Figure 4). There was no correlation between blood loss and Bb fragment levels. This is in keeping with previous studies [31] and may be clinically relevant. However, we did not observe organ dysfunction such as renal failure or pulmonary dysfunction requiring ventilatory support in this cohort of patients. Overall, due to the complex interactions of patient factors, surgical factors and the coagulation and complement activation, it is challenging to interpret these findings.

The complement and coagulation systems are closely linked. Thrombin can activate complement C3 and C5 independently [32]. Additionally, coagulation factors FIXa, FXa, FXIa and plasmin can generate C3a and C5a with cleavage of C3 and C5 independent of the known complement activation pathways [11]. Furthermore, factor XIIa has been shown to activate the classical complement pathway via activation of C1qrs complex [33], which may be highly relevant in patients undergoing CPB due to contact activation by the circuit. Therefore, inhibition of complement activation may be a therapeutic intervention to reduce post‐operative blood loss in patients undergoing CPB. Since all patients received tranexamic acid as a haemostatic agent, individual variation in the post‐operative chest tube is not attributable to use of tranexamic acid. Additionally, all patients were managed using the same institutional haemostatic management protocol.

Anaphylatoxins C3a and C5a were both significantly elevated during CPB. This corroborates previous findings by Chenoweth and associates who showed C3a generation 10 min post‐initiation of CPB surgery [7, 34]. The data suggest the generation of anaphylatoxins may be dependent in contact of the blood with foreign surfaces of the bypass machine during cardiac surgery. Additionally, it is important to highlight that our circuits were softline coated instead of heparin. C3a, generated by C3, is activated via any one of the three complement activation pathways. Therefore, no conclusions can be drawn concerning specific pathway activation. As expected, Bb fragment levels increased (Figure 1E) during the CPB suggesting alternative pathway is also activated. In addition, SC5b‐9 (Figures 1F and 2F) as well as C5a (Figures 1D and 2D) levels were raised which are both indicators of terminal complement complex activation after the initiation of CPB.

In the present study, although the magnitude of change is very small, there was a significant increase in C3a levels observed 30 min after the administration of protamine sulphate (Figures 1C and 2C). This result is in keeping with previous findings indicating that the complement system is particularly induced by heparin–protamine complexes formed following protamine sulphate administration [25]. This activation has been closely linked with the classical immune cascade [29,33]. Specifically, in vitro data have shown that the combination of heparin with protamine sulphate elevates C3a and C4a levels, indicating classical pathway activation [21]. C3 and C4 levels did not change following protamine sulphate administration (Figure 1A,B). This observation is consistent with limited data suggesting that slow IV protamine sulphate administration does not result in additional consumption of C3 and C4 despite the classical pathway being activated [5, 35].

Anaphylatoxins (C3a, C5a) and terminal complement complex levels (SC5b‐9) levels remained unchanged 30 min post‐administration of UFH but increased during surgery (Figure 1C,D,F). However, the level of Bb fragment significantly reduced 30 min post‐heparin administration (Figure 1E), a probable indication of heparin's anti‐complement action. Before protamine sulphate administration Bb levels increased, reaching its peak concentrations and 15 min after the administration of protamine sulphate levels fell significantly (Figure 1E). This fall in Bb is likely due to the loss of CPB effect (via the alternative pathway) rather than an effect of the protamine sulphate forming complexes with the heparin and activating the classical pathway. This would be consistent with the extremely short half‐life of heparin‐protamine sulphate complexes. Due to complex interaction of UFH, which may reduce the complement activation and surgical stimuli that increase the complement activation, it is difficult to differentiate the action of these two individual components separately during CPB.

Throughout bypass surgery, blood is considerably diluted, which may influence complement marker levels, although results were corrected for dilution factor using the total protein/albumin ratio. This is considered a limitation in this study and needs to be considered when interpreting the results. This is reflected in the significant increase in the total protein/albumin ratio pre‐ and post‐heparin sampling, which did return to baseline (pre‐heparin) 24 h post‐CPB. However, there were no differences in the total protein/albumin ratio sample points between post‐heparin and pre‐protamine sulphate, pre‐protamine sulphate and post‐protamine sulphate, post‐protamine sulphate and 12 h post‐CPB or 12 h post‐CPB and 24 h post‐CPB (Figure 4). Additionally, sample size is relatively small, and the study group is confined to very specific cardiac surgeries (ASD and mitral replacement). There are several studies assessing the use of pexelizumab a humanized, monoclonal, single‐chain antibody fragment that inhibits C5 in various cardiac interventions. Only two assessed the effect of pexelizumab on blood loss following coronary artery bypass graft (CABG) [29, 30], and these demonstrated a reduction in post‐operative blood loss in patients treated with pexelizumab consistent with our results. Other studies assessed the efficacy and safety of pexelizumab in reducing perioperative myocardial infarction or mortality in patients undergoing CABG with variables outcomes [26, 27, 28]. The current study provides more detailed description of changes of complement components and their activation markers during CPB and fresh review of the potential therapeutic option of the complement inhibition to reduce the post‐operative blood loss in patients undergoing CPB.

In conclusion, this study confirms that during CPB, the complement immune system is significantly activated via both the classical and alternative pathways, reaching a peak by the end of surgery. Due to complex interactions between UFH, CPB, surgical intervention and the protamine sulphate, establishing an independent effect of these factors on complement components and activation markers was challenging and requires further work. There was no correlation with complement components or activation markers with heparin levels or duration of the CPB. None of the patients developed renal failure or other organ dysfunction requiring additional support. Classical pathway complement showed inverse correlation, whilst anaphylatoxins and terminal complement makers showed positive correlation with post‐operative blood loss as measured in drain volumes. This is clinically relevant, and the inhibition of complement activation may be a therapeutic intervention in reducing post‐operative blood in patients undergoing CPB.

## FUNDING INFORMATION

DJA is funded by MRC UK (MR/V037633/1). Study was supported by Haematology Research Charity fund from Royal Brompton Hospital.

## CONFLICT OF INTEREST

The authors declare that the research was conducted in the absence of any commercial or financial relationships that could be construed as a potential conflict of interest.

## AUTHOR CONTRIBUTIONS

Rengina Kefalogianni performed laboratory assays, interpreted the data and contributed to writing the first draft of the manuscript. Farah Kamani and Jackie Donovan performed laboratory assays. Mike Laffan contributed to the data interpretation, writing and revision of the manuscript. Matthew C. Pickering contributed to the analysis of data, data interpretation and writing and revision of the manuscript. Deepa J. Arachchilage was involved in the study design, conducting the study, patient selection, laboratory assays, analysis and interpretation of the data, writing and revision of the manuscript. All the authors reviewed and approved the final manuscript.
